# Supplementary material for: Cyanobacterial Blooms in City Parks: A Case Study Using Zebrafish Embryos for Toxicity Characterization
Source: Microorganisms. 2024 Oct 2;12(10):2003. doi: 10.3390/microorganisms12102003 (PMC11509529; doi:10.3390/microorganisms12102003)
Supplement: Supplementary file 1 [file microorganisms-12-02003-s001.zip › microorganisms-3219964-supplementary.pdf]

Supplemental material

Cyanobacterial blooms in city parks: a case study using zebrafish embryos for toxicity characterization

Bruna Vieira [1], João Amaral [1], Mário Jorge Pereira [2], Inês Domingues [2]

[1] Department of Biology, Campus Universitário de Santiago, 3810-193 Aveiro, Portugal; [2] Department of Biology & CESAM, , Campus Universitário de Santiago, 3810-193 Aveiro, Portugal

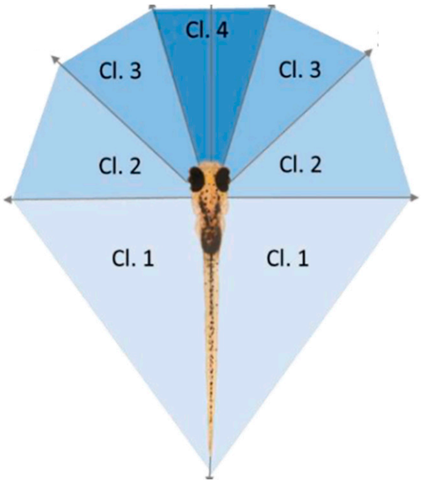

Figure S1- Schematic representation of larvae path angles and grouped classes considered on the behaviour analysis. Adapted from Santos et al., (2021).

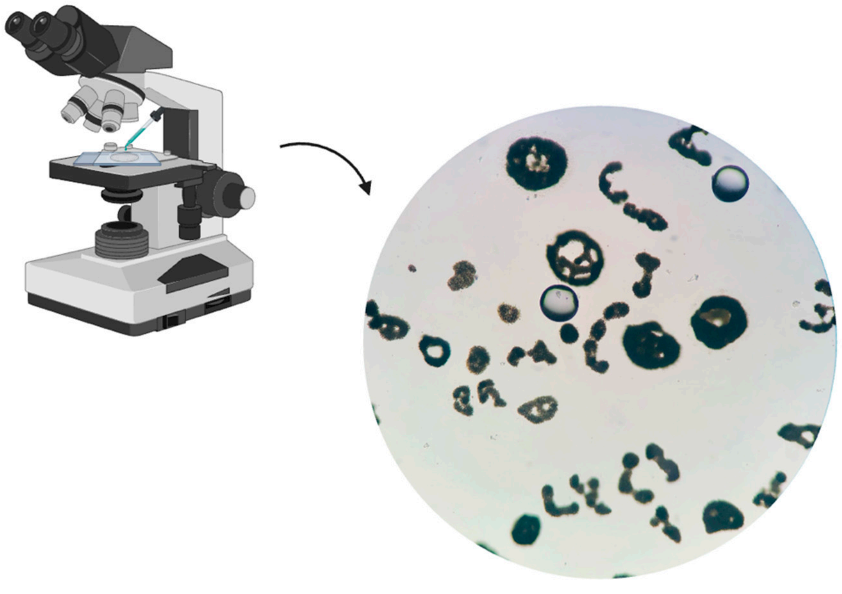

Figure S2 – *Microcystis aeruginosa*. Image obtained from the optical microscope observation of the samples collected.
